# Cyclosporine A-Nanosuspension: Formulation, Characterization and *In Vivo* Comparison with a Marketed Formulation

**DOI:** 10.3797/scipharm.0908-12

**Published:** 2010-04-26

**Authors:** Mahendra Nakarani, Priyal Patel, Jayvadan Patel, Pankaj Patel, Rayasa S. R. Murthy, Subhash S. Vaghani

**Affiliations:** 1 Unison Pharmaceuticals, Ahmedabad, Gujarat, India; 2 S. K. Patel college of Pharm Edu & Res, Ganpat University, Gujarat, India; 3 Nootan pharmacy college, Visnagar, Gujarat, India; 4 TIFAC-CORE in NDDS, The M.S.University of Baroda, India; 5 Smt. R. B. Patel Mahila Pharmacy College, Atkot, India

**Keywords:** Cyclosporine A, Nanosuspension, Scanning electron microscopy, Poloxamer, Osmolarity

## Abstract

Cyclosporine A-nanosuspensions were prepared using zirconium oxide beads as a milling media, Poloxamer 407 as a stabilizer and distilled water as an aqueous medium using the Pearl Milling technique. The optimized formulation was characterized in terms of particle size distribution, surface morphology, drug-surfactant interaction, drug content, saturation solubility, osmolarity, and stability. The nanoparticles consisting of Poloxamer-bound cyclosporin A with a mean diameter of 213 nm revealed a spherical shape and 5.69 fold increased saturation solubility as compared to the parent drug. The formulation was found to be iso-osmolar with blood and stable up to 3 months at 2–8°C. *In-vivo* studies were carried out in albino rats and the pharmacokinetic parameters were compared with a marketed formulation, which indicated better results of the prepared formulation than the marketed one.

## Introduction

Cyclosporine A, a neutral hydrophobic cyclic peptide composed of 11 amino acid residues, is a 3^rd^ generation immunosuppressant, used in organ transplantation since 1981 [1, 2]. The aqueous solubility of CsA is very low and it displays a considerable inter and intra patient variability presumably due to its poor and highly bile dependent absorption as well as intestinal metabolism. Hence therapy requires careful monitoring of blood levels [3]. Exceeding the therapeutic window adverse effects like nephrotoxicity and hepatotoxicity are reported. Furthermore it has been reported that at sub therapeutic drug levels, organ transplantation rejection occurs after systemic drug administration in case of lung, heart-lung or corneal transplantation [4].

Major efforts have concentrated on development of customized drug carriers to overcome the disappointing *in vivo* characteristics of the drug. For carriers non-toxicity (acute and chronic), sufficient drug loading capacity, possibility of drug targeting, controlled release characteristics, chemical and physical storage stability (for both drug and carrier) and feasibility of scaling up production with reasonable overall costs are requested [5–7]. Colloidal carriers have attracted the main interest because they are promising systems to fulfill the requirements mentioned above. But in the first place, nanosized carriers are treated as a hopeful means to increase the solubility and therefore the bioavailability of poorly water-soluble active ingredients belonging to the classes II and IV in the Biopharmaceutical Classification System (BCS) [8–10]. Scaling up production at reasonable overall costs are requested. In conventional dosage forms of CsA, available at the market as sandimmune®, Cremophor EL (polyoxyethylated castor oil) is used as a solubilizer associated with the major problems of Cremophor EL such as anaphylactic shock. In addition vascular collapse due to nephrotoxicity and hemolysis as well as respiratory distress related release of histamine can occur as side-effects [3, 11].

Recently, it has been found that the majority of the water insoluble drugs belong to the group of anti-cancer agents, anti-infectiva, central nervous system (CNS) and anti-viral therapeutics. With the increasing incidence of these diseases, there has been a strong interest in nanosuspension dosage forms for injectable applications evolved. Following the approval of a nanoparticulate i.v dosage form, Abraxane® (130 nm amorphous particles of paclitaxel entrapped in human serum albumin), in 2005, there has been a steady increase in the drug nanoparticulate formulations entering clinical trials [12]. An important advantage of the drug nanosuspensions is their administration via various routes, such as oral [13], parenteral [14], ocular [15] and pulmonary delivery [16]. In addition, nanosuspensions have been shown superior to their counterparts of formulated traditionally in every administration route. For nanosuspensions, according to Noyes–Whitney and Ostwald–Freundlich equation, particle size in nanometer range can lead to increased dissolution velocity and saturation solubility [17, 18] so that oral absorption of poorly soluble drugs together with higher bioavailability can be achieved as compared to the traditional formulation [13,19]. Beyond that, due to their sufficiently small size and safe composition, nanosuspensions can be injected intravenously, and 100% bioavailability can be reached [20].

The present work is carried out to overcome the toxicity caused by Cremophore EL 35 that is currently used in conventional formulations and to minimize, the P-Glycoprotein mediated drug- efflux by developing a stable nanosuspension of CsA.

## Results and Discussion

### Particle size and Size distribution

**Fig. 1.**
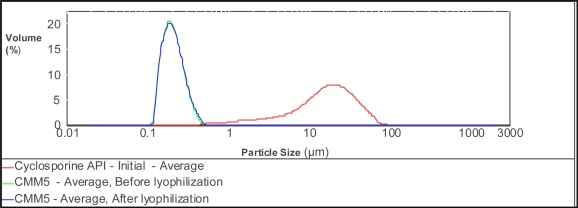
Size distribution of the optimized batch (CMM 5) of CsA-nanosuspensions.

The optimized batch (CMM 5) had a mean particle diameter [d(4,3)] of 213nm with uniformity (absolute deviation from median value) 0.245 (before lyophilization) with 2.5%w/v of stabilizer and 40%v/v particles. After lyophilization the mean particle diameter was still 216nm with uniformity 0.25 (Table 1, 2). Increasing the media volume led to slightly higher particle diameters but did not significantly change by increasing the concentration of stabilizer. The lowest mean particle diameter was obtained at 8h milling in 50:50 ratio of milling media. Further stirring ncreased the mean particle diameter of the CsA nanosuspension. The resulting nanosuspensions were of uniform particle size in the range around 200nm, which is very important for *in vivo* biodistribution. The particle size distribution pattern of the optimized nanosuspension formulation is given in figure 1.

The comparison of the bioavailability of CsA from solid lipid nanoparticles and nanocrystals revealed low variation in bioavailability from SLN as compared to nanocrystals of CsA [21]. Poloxamers appear to exert their effects on P-glycoprotein via direct or indirect inhibition, on membrane fluidity, adenosine triphosphate (ATP), depletion or on osmolarity [22].

### Scanning electron microscopy (SEM)

Scanning electron images reveal a change in appearance of the surface upon formulating the nanosuspension. The altered shape might be due to coating of CsA-particles with a surfactant/stabilizer layer and creation of an amorphous surface layer due to the high attrition and shearing rate [23].

### Differential Scanning Calorimetry (DSC)

**Fig. 3.**
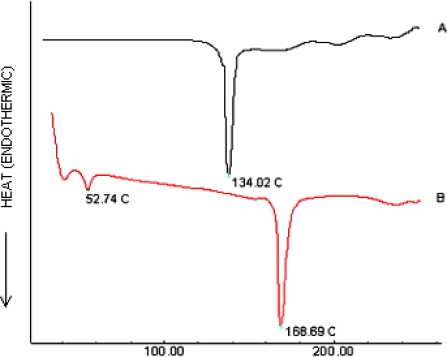
DSC thermograms of (A) Plain drug (CsA) and (B) CsA-nanosuspension

The DSC thermograms of Plain drug (CsA) and optimized nanosuspension formulation were taken on a Mettler DSC 20 differential scanning colorimeter between 30–200°C at a heating rate of 20°C/min. Pure CsA showed melting point at 134.02°C corresponding to its melting point [24], whereas in the thermograph pattern of formulation no such peak was observed (figure 3) [25]. So it can be concluded that the drug particles were absolutely bound by the surfactant molecules [26]. As shown in figure 3 in the DSC of formulation sharp transitions at 54.72°C and 168.69°C were observed which correspond to the melting points of (poloxamer) [27] and (mannitol) respectively [28].

### pH

For intravenous dosage form the pH should be within physiological pH range. Table 3 shows the pH of the nanosuspension before and after addition of a cryoprotectant (1:1 ratio). It was observed that it was in range.

### Saturation solubility

The saturation solubility is equilibrium between dissolving molecules (dissolution pressure) and re-crystallizing molecules [29]. The saturation solubility increases with decreasing particle size according to the Ostwald–Freundlich equation [30].

The results of saturation solubility of plain drug (CsA) and lyophilized powder of the CsA-nanosuspension (see table 3) revealed a saturation solubility of 34.59μg/ml (plain CsA) and 196.94μg/ml (CsA-nanosuspension). Thus saturation solubility of CsA as a nanosuspension is 5.69 fold higher than that of plain CsA. In the present study, particle size of CsA has been reduced hence increased the saturation solubility due to increasing the surface area of the reduced particles [31].

### Drug content [32]

In nanosuspension formulation the drug particles were reduced to nano sized. During the formulation process there was not any drug loss step involved, so theoretically the formulation was considered as being 100% drug content. As shown in Table 3 the drug content was found to be 99.12%w/w.

### Osmolarity

Intravenous dosage form should be isoosmolar with the blood (250–350mOsmol/L) so the optimized nanosuspension formulation was checked for osmolarity and results revealed that prepared nanosuspension was isoosmolar with the blood (Table 3).

### Stability Studies

**Fig. 4.**
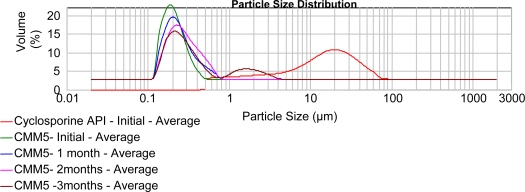
Size distribution of the above optimized batch of nanosuspension during storage at 2–8 °C.

During storage as aqueous dispersions, nanosuspensions sometimes show physical instability cause by aggregation. However, crystal growth does not play a role in storage of nanosuspensions at room temperature or at 5°C over a period of months. To avoid aggregation, formulation should be transferred into a dry product, such as by lyophilization or spray-drying. Nanosuspensions can be easily lyophilized and are well re-dispersible [18]. Stability study of nanosuspension formulation was carried out at 2–8°C. The optimized batch was stable up to 3 months with slightly increased in the particle size on storage up to 3 months (Table 2). The particle size distribution indicated uniformity initially but due to the storage the particles aggregation might have occurred and so particle size was increased and uniformity was reduced (Figure 4).

### In vivo study [32–35]

According to one study, in which a 14-day studies of intravenous administration of poloxamer at concentrations up to 0.5 g/kg/day to rabbits was performed and no overt adverse effects were noted. A similar study with dogs also showed no adverse effects at dosage levels of poloxamer up to 0.5 g/kg/day [28].

**Fig. 5.**
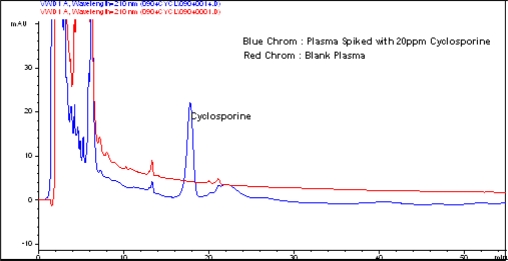
Overlaid spectrum of CsA spiked in plasma and blank plasma

**Fig. 6.**
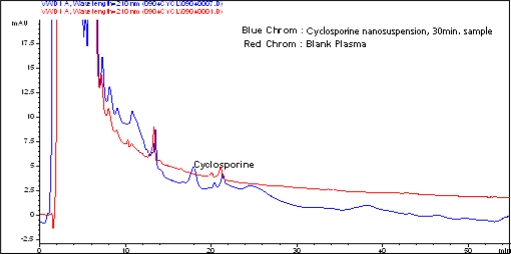
Overlaid spectrum of CsA detected in plasma samples of the nanosuspension and blank plasma

*In vivo* study was performed for estimation of cyclosporine in plasma. Figure 5 shows an overlaid spectrum of cyclosporine spiked in plasma and blank plasma, and figure 6 shows an overlaid spectrum of cyclosporine detected in plasma sample of nanosuspension and blank plasma.

**Fig. 7.**
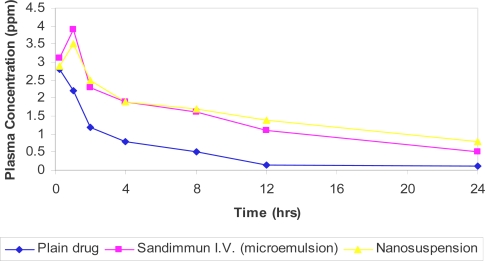
Mean blood concentration-time profiles of CsA following intravenous administration

Figure 7 shows the mean blood concentration versus time profiles after intravenous administration of CsA in three different dosage forms namely: free drug, marketed microemulsion formulation (Sandimmune® I.V.) and nanosuspension formulation. The amount of drug detected in the plasma of different rats at different time points along with standard deviation is as shown in the table 4.

A peak concentration was reached within 1 hour in all the three formulations but the maximum concentration at 1 hour was observed with the marketed formulation (table 4). There was no significant difference between the nanosuspension and Microemulsion formulation (marketed formulation). The half life of CsA from the intravenously administered nanosuspension formulation was greater than the microemulsion formulation (Table 5).

The difference between the nanosuspension and marketed solution was statistically non significant (p < 0.05). The difference between the free drug and the marketed solution was statistically significant (p > 0.05). In addition, the difference between the free drug and the nanosuspension was statistically significant (p > 0.05). The elimination rate constant (K_el_) was lower for nanosuspension formulation than the other two formulations. Based on this work, it is believed that an alternative Nanosuspension formulation for intravenous administration can be obtained from which the cyclosporine can exert its clinical effects with minimum inter individual variability. From the figure 7 and table 4 and 5 we can say that the prepared nanosuspension is equal to that of marketed preparation.

## Experimental

### Materials

Cyclosporine was obtained as a gift sample from RPG Lifesciences Ltd. Ankleshwar, India; Poloxamer 407 was obtained from BASF, Germany; Zirconium Oxide beads were obtained as gift samples from Sun Pharmaceutical Industries Ltd., India; Mannitol, Methanol and Tetrahydrofuran were purchased from S.D. Fine Chemicals, India; Acetonitrile (HPLC Grade) and Methanol (HPLC Grade) were obtained from Spectrochem Pvt. Ltd., India.

### Methods

#### Preparation of Cyclosporine based Nanoparticles

An attempt was made to prepare stable nanosuspension of Cyclosporine A (CsA) using Zirconium Oxide beads as a milling media, Poloxamer 407 as a stabilizer, distilled water as an aqueous medium, using Pearl milling technique. The various parameters like effect of stirring time, stirring speed and ratio of beads were optimized by keeping the drug: Surfactant: Milling media (1:3.0:50) as constant initially and the optimized condition was used throughout the study (Tables 6 and 7). These parameters were optimized at 25°C. Milling equipment was cleaned using mild soap solution first and after that several washes with deionised water were made to remove all traces of detergent. The final wash was carried out with distilled water.

Nanosuspension containing cyclosporine was optimized for formulation parameters by 3^2^ factorial designs. Nanosuspensions were prepared by dissolving different concentration of surfactant (Poloxamer 407) in distilled water then drug was added directly into the surfactant solution and comminuted using Zirconium Oxide beads on a magnetic stirrer. The process and formulation parameters were optimized to achieve minimum particle size. The coded values and observations of the optimization process by 3^2^ factorial designs are tabulated in table 8.

### Lyophilization method

The optimized nanosuspension (CMM-5) was lyophilized using mannitol (1:1 ratio) as a cryoprotectant. Nanosuspension containing ampoules were freeze in deep freezer at – 20°C for 8h (EIE, India) for primary freezing. The ampoules were then transferred to flask and the flask was attached to the vacuum adapter of lyophilizer (hetodry winner). The solvent was sublimed under a pressure of 80 mmHg for 24h.

### Particle size and Size distribution

The mean particle size and size distribution of the prepared nanosuspension formulations were obtained by using Malvern particle size analyzer SM 2000, which followed Mies theory of light scattering. Particle size detection range for Malvern SM 2000 is 0.02 to 2000 μm. Nanosuspensions were added to the sample dispersion unit containing stirrer and stirred at 2000rpm in order to reduce the interparticle aggregation, and laser obscuration range was maintained between 10–20%. The average particle sizes were measured after performing the experiment for each batch in triplicate.

### Scanning electron microscopy (SEM)

The lyophilized powder of nanosuspension formulation was kept in the sampling unit as a thin film and then photographs were taken at 100X & 400X magnification using Scanning Electron Microscope (Jeol, JSM-840 SEM Japan).

### Differential Scanning Calorimetry (DSC)

The DSC thermograms of Plain drug (CsA) and optimized nanosuspension formulation were taken on a Mettler DSC 20 differential scanning colorimeter between 30–200°C at a heating rate of 20°C/min. The thermograms of Plain drug (CsA) and optimized nanosuspension formulation are shown in figure 3.

### pH

Prepared nanosuspension was taken in 10ml beaker and pH was measured using pH meter (Digital Instrument Corporation, India). Table 3 shows the pH of the nanosuspension before and after addition of cryoprotectant.

### Saturation solubility

Saturation solubility is a compound-specific constant only depending on the temperature and the properties of the dissolution medium. However, below a size of approximately 1–2 μm, the saturation solubility is also a function of the particle size [29].

Saturation solubility of Plain drug (CsA) and lyophilized powder of optimized nanosuspension formulation were carried out in phosphate buffer pH 7.4 for which 5mg of drug and lyophilized powder (weigh equivalent to 5mg of drug) in 2ml phosphate buffer pH 7.4 were taken separately and were allowed to be stirred in an isothermal shaker (37.0 ± 1.0°C) for 24h. The stirred samples were further taken in test tubes and centrifuged (Remi) at 10000 rpm for 15 minutes. The supernants were collected, diluted 5 times with PBS pH 7.4 and absorbance were measured at 210nm [32] using UV-Visible spectrophotometer. The solubility was measured at 25°C.

### Drug content

Drug content of nanosuspension formulation was carried out by taking lyophilized powder (weigh equivalent to 5mg of drug) in methanol:THF (1:1) mixture, shaken well, Mannitol is slightly soluble in methanol:THF (1:1) mixture so it was then centrifuged at 8000rpm for 10min. The supernatants were taken and diluted with methanol:THF (1:1) mixture and the absorbance was measured at 210nm. The drug content was calculated using the calibration curve. The results are shown in Table 3.

### Osmolarity

Practically Osmolarity was measured using Osmometer and theoretically it was calculated using following formula:

$$\text{Osmolarity}\;(\text{mOsmol}/\text{L})=\frac{\text{Weight}\;\text{in}\;\text{gm}/\text{lit}\times \text{No}.\text{of}\;\text{species}\times 1000}{\text{Molecular}\;\text{weight}\times 100}$$

### Stability study

Stability of optimized nanosuspension formulation containing cyclosporine was evaluated by determining change in particle size during storage at 2–8°C. Any change in particle size of nanosuspension formulation was observed using Malvern Mastersizer 2000 at periodic time intervals.

### In vivo study [33]

Three groups of two albino rats were taken for the study. The study was carried out at the Mercury Laboratories, Vadodara. All experiments and protocols described in this study were approved by the Institutional Animal Ethics Committee of MS University of Baroda and are in accordance with the Committee for Purpose of Control and Supervision of Experiments on Animals (CPCSEA), Ministry of Social Justice and Empowerment, Government of India with the permission number 404/01/a/CPCSEA. The rats were fasted overnight and then each group was given a different cyclosporine formulation viz. free drug, marketed sample of cyclosporine namely Sandimmune® I.V. (Microemulsion) and Nanosuspension (Table 9). The normal human dose of Cyclosporine is 5 mg/kg of body weight. This dose was converted into animal dose by using the formula given below:-

$$\text{HED}=\;\text{Animal}\;\text{dose}\;\text{in}\;\text{mg}/\text{kg}/{\left(\text{animal}\;\text{body}\;\text{weight}\;\text{in}\;\text{kg}/\text{human}\;\text{body}\;\text{weight}\;\text{in}\;\text{kg}\right)}^{1/3}$$

Where HED =Human equivalent dose
Animal body weight = 0.320kg
Human body weight = 60kg

By using this formula, dose of Cyclosporine given to rats was obtained 300μg. Hence, each of 3 formulations was administered at the dose of 300µg Cyclosporine. The blood samples were collected at 30min, 1h, 2h, 4h, 8h, 12h and 24h following I.V. administration. 100μl of plasma was taken in a 0.5ml micro centrifuge tube and 100μl of acetonitrile was added into it to precipitate the proteins present in plasma. The tube was vortexed for 5 min followed by centrifugation at 15000 rpm for 10 min. The supernatant was taken and 50μl was injected into Shimadzu-AS HPLC system. The calibration curve of plasma spiked with cyclosporine was taken in a range of 0.06 to 50 ppm. Data analysis was done using QUICK CALC software. Statistical analysis was done using a two tail paired t-test for determining statistical significance (p < 0.05). All results were expressed as the mean ± standard deviation (S.D.).

## Conclusion

From the above results we have concluded that the present investigation of nanosuspension formulation containing CsA can be an alternative dosage form of intravenous microemulsion formulation by avoiding a hypersensitivity reaction caused by Cremophore EL 35. All Pharmacokinetic parameters show favorable results. It can modulate the disposition of drug in the body and potentially improve the safety & efficacy profile of drug. It is a simple, cost effective and scalable method as compared to other methods.

## Figures and Tables

**Fig. 2.**
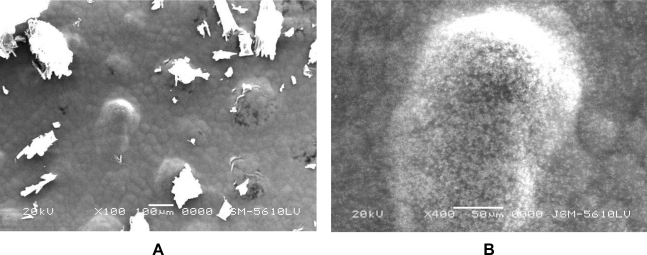
Scanning electron image of CsA-nanosuspension CMM 5 at (A) 100X and (B) 400X magnification.

**Tab. 1.** Optimization of the parameters for the preparation of CsA-nanosuspensions

**Batch No.**	**Conc. of drug (% w/v)**	**Conc. Surfactant (Poloxamer 407)**	**% v/v of Milling Media (ZrO_2_ beads)**	**Particle size before Lyophillizatio n [d(4,3)]**	**Uniformity**	**Particle size after Lyophillization [d(4,3)]**	**Uniformity**
CMM-1	1	3.0	50	0.227μm	0.260	0.237μm	0.289
CMM-2	1	3.0	40	0.217μm	0.253	0.235μm	0.284
CMM-3	1	3.0	60	0.262μm	0.361	0.351μm	0.663
CMM-4	1	2.5	50	0.294μm	0.369	0.565μm	1.33
CMM-5	1	2.5	40	0.213μm	0.245	0.216μm	0.250
CMM-6	1	2.5	60	0.737μm	1.98	1.242μm	5.43
CMM-7	1	3.5	50	0.263μm	0.365	1.868μm	6.24
CMM-8	1	3.5	40	0.214μm	0.247	0.225μm	0.256
CMM-9	1	3.5	60	0.199μm	0.223	0.212μm	0.246

**Tab. 2.** Particle diameter of the optimized batch (CMM 5) of lyophilized nanosuspension during storage at 2–8°C.

**Time (Months)**	**Mean Particle size [d(4,3)]**	**Uniformity**
Initial	0.217μm	0.260
1	0.268μm	0.372
2	0.320μm	0.375
3	0.600μm	1.98

**Tab. 3.** Results of pH, Saturation solubility, Assay and Osmolarity of cyclosporine nanosuspension formulation.

**Sr. No.**	**Parameters**		**Results**
1.	pH	With Cryoprotectant (Mannitol)	7.49
		Without Cryoprotectant (Mannitol)	6.74
2.	Saturation solubility	Plain drug (CsA)	34.59μg/ml
		CsA nanosuspension	196.94μg/ml
3.	Drug content		99.12%w/w
4.	Osmolarity	Practically determined	320.20mOsmol/L
		Theoretically calculated	317.44mOsmol/L

**Tab. 4.** Blood cyclosporine A concentration in three groups of rats receiving different formulations.

**Product**	**Time (h)**	**Concentration (μg/ml)**		**Mean ± S.D**
		**1**	**2**	
Free drug	0.30	2.8	2.9	2.85 ± 0.070
	1	2.2	2.3	2.25 ± 0.070
	2	1.2	1.1	1.15 ± 0.070
	4	0.8	0.6	0.70 ± 0.141
	8	0.5	0.4	0.45 ± 0.070
	12	0.15	0.13	0.14 ± 0.141
	24	0.1	0.09	0.09 ± 0.070

Marketed sample (Sandimmune® I.V.)	0.30	3.1	3.13	3.10 ± 0.021
	1	3.9	4.1	4.00 ± 0.141
	2	2.3	2.1	2.20 ± 0.141
	4	1.9	1.8	1.85 ± 0.070
	8	1.6	1.4	1.50 ± 0.141
	12	1.1	0.9	1.00 ± 0.141
	24	0.5	0.4	0.45 ± 0.070

Nanosuspension	0.30	2.9	3.1	3.00 ± 0.141
	1	3.5	3.6	3.55 ± 0.070
	2	2.5	2.4	2.45 ± 0.070
	4	1.9	1.7	1.80 ± 0.141
	8	1.7	1.6	1.65 ± 0.070
	12	1.4	1.1	1.25 ± 0.212
	24	0.8	0.7	0.75 ± 0.070

**Tab. 5.** Mean pharmacokinetic parameters of CsA following intravenous administration of three dosage forms (dose 300μg per rat)

	**Free drug**	**Sandimmune® I.V. (Microemulsion)**	**Nanosuspension**
C_max_ (μg/ml)	2.8	3.9	3.5
T_1/2_ (h)	2.01	6.867	9.88
K_el_ (ml/min/kg)	0.344	0.100	0.07
V_d_	100.49	88.353	97.21
Total Cl	34.58	8.91	6.89
AUC	8.67	33.64	44.01

**Tab. 6.** Effect of stirring time for the preparation of nanosuspension containing cyclosporine

**Batch. No.**	**Time (h)**	**Mean particle size [D(4,3)]**
ST1	Initial (5min.)	19.95μm
ST2	2	2.739μm
ST3	4	0.884μm
ST4	6	0.526μm
ST5	8	0.230μm
ST6	10	0.238μm
ST7	12	0.242μm

**Tab. 7.** Effect of Ratio of beads for the preparation of nanosuspension containing cyclosporine

**Batch. No.**	**Ratio of beads (Zirconium Oxide)**		**Mean particle size [D(4,3)]**
	**Small Size (0.4–0.7mm)**	**Big Size (1.2–1.7mm)**	
RB1	100	0	0.435μm
RB2	75	25	0.364μm
RB3	50	50	0.237μm
RB4	25	75	0.534μm
RB5	0	100	0.685μm

**Tab. 8.** 3^2^ factorial design layout for optimization of nanosuspension containing cyclosporine.

**Batch No.**	**X_1_**	**X_2_**
CMM-1	3.0	50
CMM-2	3.0	40
CMM-3	3.0	60
CMM-4	2.5	50
CMM-5	2.5	40
CMM-6	2.5	60
CMM-7	3.5	50
CMM-8	3.5	40
CMM-9	3.5	60

**X_1_** – Concentration of Surfactant (Polaxamer 407) (% w/v)

**X_2_** – % v/v of Milling Media (Zirconium oxide beads)

**Tab. 9.** Different formulation and volume administered to different groups of animals.

**Animal No.**	**Formulation**	**Body weight (gm)**	**Volume to be injected(ml)**
1	Free drug	320	0.28
2		338	0.30
1	Microemulsion (Sandimmune® I.V.)	318	0.28
2		326	0.29
1	Nanosuspension	340	0.30
2		332	0.29
